# Structural basis of the dominant inheritance of hypermethioninemia associated with the Arg264His mutation in the *MAT1A* gene

**DOI:** 10.1107/S2059798320006002

**Published:** 2020-05-29

**Authors:** Jiraporn Panmanee, Svetlana V. Antonyuk, S. Samar Hasnain

**Affiliations:** a Molecular Biophysics Group, Institute of Systems, Molecular and Integrative Biology, Faculty of Health and Life Sciences, University of Liverpool, Crown Street, Liverpool L69 7ZB, United Kingdom

**Keywords:** methylation, *S*-adenosylmethionine, hypermethioninemia, drug discovery

## Abstract

Methionine adenosyltransferase (MAT) deficiency, characterized by isolated persistent hypermethioninemia (IPH), is caused by mutations in the *MAT1A* gene encoding MATαl, one of the major hepatic enzymes. Most of the associated hypermethioninemic conditions are inherited as autosomal recessive traits; however, dominant inheritance of hypermethioninemia is caused by an Arg264His (R264H) mutation. This mutation has been confirmed in a screening programme of newborns as the most common mutation in babies with IPH. Arg264 makes an inter-subunit salt bridge located at the dimer interface where the active site assembles. Here, it is demonstrated that the R264H mutation results in greatly reduced MAT activity, while retaining its ability to dimerize, indicating that the lower activity arises from alteration at the active site. The first crystallographic structure of the apo form of the wild-type MATαl enzyme is provided, which shows a tetrameric assembly in which two compact dimers combine to form a catalytic tetramer. In contrast, the crystal structure of the MATαl R264H mutant reveals a weaker dimeric assembly, suggesting that the mutation lowers the affinity for dimer–dimer interaction. The formation of a hetero-oligomer with the regulatory MATβV1 subunit or incubation with a quinolone-based compound (SCR0911) results in the near-full recovery of the enzymatic activity of the pathogenic mutation R264H, opening a clear avenue for a therapeutic solution based on chemical interventions that help to correct the defect of the enzyme in its ability to metabolize methionine.

## Abstract 2

Dominant inheritance of hypermethioninemia is caused by an Arg264His mutation in methionine adenosyltransferase. The mutation lowers the affinity for dimer–dimer interaction and enzymatic activity, which can be restored by chemical intervention.

## 1. Introduction   

Methionine adenosyltransferase (MAT) deficiency (OMIM 250850) is the most common cause of isolated persistent hypermethioninemia (IPH), which has been established as an inborn error of metabolism (Gaull & Tallan, 1974 ▸). MAT enzymes (EC 2.5.1.6) catalyse the formation of *S*-adenosyl-l-methionine (SAMe) from methionine (Met) and adenosine triphosphate (ATP). MATs are found in all living organisms, with the exception of some intracellular parasites that obtain SAMe from their host (Sánchez-Pérez *et al.*, 2004 ▸). Normal cellular function and survival require SAMe as a versatile molecule with roles ranging from gene expression to membrane fluidity (Finkelstein, 1990 ▸; Friedel *et al.*, 1989 ▸; Lu, 2000 ▸; Mato *et al.*, 1997 ▸). In mammalian cells, MATα1 and MATα2 are two variants of the catalytic subunit encoded by the *MAT1A* and *MAT2A* genes, respectively (Chamberlin *et al.*, 1996 ▸; De La Rosa *et al.*, 1995 ▸). MATα1 has 84% amino-acid sequence similarity to MATα2, despite their distinct kinetic properties and expression in distinct tissues. MATα1 is a liver-specific enzyme that is mainly found in mature hepato­cytes, while MATα2 is expressed in extrahepatic tissues including kidney, brain and heart and also in developing liver cells (Alvarez *et al.*, 1993 ▸; Horikawa & Tsukada, 1992 ▸; Kotb & Geller, 1993 ▸). Patients with IPH have low levels of hepatic MAT (MATα1) activity, resulting in high levels of methionine in the blood plasma. Case reports of MATα1 deficiency were identified through newborn screening programmes where the activity of MATα2 in erythrocytes, lymphocytes and fibroblasts was found to be normal (Gahl *et al.*, 1988 ▸; Gaull *et al.*, 1981 ▸). Hepatic MAT deficiency is usually inherited as an autosomal recessive trait (Chamberlin *et al.*, 1996 ▸, 2000 ▸; Mudd *et al.*, 1995 ▸; Ubagai *et al.*, 1995 ▸) or as the exceptional Arg264His (R264H) autosomal dominant mutation (Chien *et al.*, 2005 ▸; Couce *et al.*, 2008 ▸, 2013 ▸; Martins *et al.*, 2012 ▸; Ubagai *et al.*, 1995 ▸). Neurological problems, including demyelination, abnormal mental development and cognitive impairment, have been reported in severe cases of autosomal recessive IPH, while the majority of patients with a mild to moderate condition present no clinical manifestations (Chamberlin *et al.*, 1996 ▸, 2000 ▸; Hazelwood *et al.*, 1998 ▸; Kido *et al.*, 2019 ▸; Tada *et al.*, 2004 ▸; Sen *et al.*, 2019 ▸). R264H is the most prevalent mutation found to cause IPH-associated MATα1 deficiency in newborn screening programmes, as reported in the USA (three of 13 cases), Spain (15 of 18 cases), Japan (14 of 24 cases), Portugal (all 12 cases) and Taiwan (three of 16 cases) (Couce *et al.*, 2013 ▸; Martins *et al.*, 2012 ▸; Nagao *et al.*, 2013 ▸; Chadwick *et al.*, 2014 ▸; Chien *et al.*, 2005 ▸). IPH related to the R264H mutation is usually clinically benign in the early stages of life (Couce *et al.*, 2013 ▸; Nagao *et al.*, 2013 ▸); however, the discovery of a three-year-old girl with myelination abnor­malities and the severe vascular diseases reported in some cases related to this mutation suggest that clinical monitoring should be performed (Martins *et al.*, 2012 ▸).

SAMe is the principal methyl donor in the transmethylation processes of important cellular biomolecules, including DNA, RNA, proteins, neurotransmitters *etc.* (Chiang *et al.*, 1996 ▸). It is also involved in the trans-sulfuration pathway related to the biosynthesis of major cellular antioxidants (for example glutathione and taurine). Furthermore, SAMe is a precursor in polyamine synthesis, which is important in biological processes including cell proliferation, cell differentiation and apoptosis. The formation of SAMe and its level are crucial in normal cellular functions that derive from its versatile roles, while its level is found to be relatively low when the methionine level is high in patients with IPH (Mudd, 2011 ▸).

The catalytic MAT isoforms are biologically active only when they assemble as a dimer or a tetramer, as the functional active sites are formed at the dimer interface. MATα1 functions as a homodimer and a homotetramer (Kotb *et al.*, 1997 ▸), while MATα2 forms a hetero-oligomer with the regulatory subunit MATβ, which has two isoforms (MATβV1 and MATβV2). MATα2 has been found to form complexes in a 2:1 ratio with the V1 and V2 isoforms of the regulatory subunit [MAT(α2)_4_(βV1)_2_ and MAT(α2)_4_(βV2)_2_] (LeGros *et al.*, 2000 ▸, 2001 ▸; Lu, 2000 ▸; Martínez-Chantar *et al.*, 2003 ▸; Murray *et al.*, 2014 ▸). In order to understand the structure–function relationship behind the dominant effect of the R264H mutation on MAT enzymes, we generated the R264H mutant of the MATα1 protein, measured its enzymatic activity and determined the oligomeric form of this mutant both in solution and in three-dimensional crystallo­graphic structures. In the central region of the wild-type MATα1 dimer the MAT subunits interact through polar contacts, creating a salt bridge between Arg264 of one subunit and Glu57 of the other. This interaction has been reported to be essential for dimerization and to also be of importance for the formation of the active site (Chamberlin *et al.*, 1997 ▸; Mato *et al.*, 2001 ▸). Our data show that the R264H mutant exists as a dimer and a tetramer in solution in almost equal amounts, in contrast to the wild-type enzyme, which exists primarily as a tetramer. The R264H mutant crystallized in the dimeric form, which provided a 2.85 Å resolution crystallographic structure. Our data suggest that the R264H mutation does not affect dimerization, but increases the propensity to form a dimer compared with the wild-type enzyme. The crystal structure was obtained in the apo form with no methionine or SAMe bound, even when we attempted to co-crystallize the R264H mutant with the substrates methionine and AMP-PNP, which was consistent with the defective nature of the catalytic machinery of the mutant enzyme. We show that the activity of the R264H mutant can be restored to the levels found for the wild-type enzyme when the mutant is incubated with the quinolone compound SCR0911 or the regulatory subunit MATβV1. This discovery opens an opportunity for chemical intervention to alleviate this disease-causing defect in catalysis.

## 2. Materials and methods   

### 2.1. Protein expression   

The MATα1 plasmid (pNIC28-Bsa4) construct was generously provided by the Oxford Structural Genomics Consortium. The MATβV1 and MATβV2 constructs were kindly provided by the laboratory of Dr Shelly Lu, University of California Los Angeles and were subcloned into pET-28M-SUMO3 vector. All plasmids were transformed into *Escherichia coli* strain BL21 (DE3). Transformed cells were plated on selective LB agar supplemented with 50 µg ml^−1^ kanamycin and grown overnight at 37°C. A single colony was used to make a starter culture. 5 ml of the freshly grown starter culture was added to 500 ml LB medium mixed with 50 µg ml^−1^ kanamycin and the cultures were shaken at 240 rev min^−1^ and 37°C until the cell density reached an OD_600_ of 0.6–0.8. Protein expression was induced by the addition of 1 m*M* isopropyl β-d-1-thiogalactopyranoside (IPTG). The cultures were grown overnight at 240 rev min^−1^ and 20°C. The cells were harvested by centrifugation at 6000*g* for 20 min at 4°C and flash-frozen using liquid nitrogen before storage at −80°C.

### 2.2. Protein purification for MATα1 and MATβV1/V2   

The cell pellets were resuspended in ice-cold lysis buffer [500 m*M* NaCl, 5%(*v*/*v*) glycerol, 5 m*M* imidazole, 10 m*M* β-mercaptoethanol (βME)] mixed with 1 m*M* phenylmethylsulfonyl fluoride (PMSF) at a ratio of 1 g pellet to 4 ml lysis buffer. For cell disruption, 25 ml cell suspension was sonicated 15 times on ice (30 s on/off cycles). The cell suspension was centrifuged at 20 000*g* for 40 min to remove cell debris. The clear supernatant was collected and loaded onto a HisTrap HP column (GE Healthcare, Chicago, Illinois, USA) pre-equilibrated with lysis buffer. The column was washed with ten column volumes of lysis buffer and then with five column volumes of wash buffer [500 m*M* NaCl, 5%(*v*/*v*) glycerol, 30 m*M* imidazole, 10 m*M* β-ME]. The proteins were eluted with elution buffer (500 m*M* NaCl, 250 m*M* imidazole, 10 m*M* β-ME). To cleave the His tag from MATα1, the eluted fractions containing the protein were pooled together and incubated with Tobacco etch virus (TEV) protease at a 100:1 (protein:TEV protease) ratio followed by dialysis against dialysis buffer (25 m*M* HEPES pH 7.5, 250 m*M* NaCl, 10 m*M* β-ME) overnight at 4°C. To cleave the SUMO tag from MATβV1 and MATβV2, the eluted proteins were incubated with sentrin-specific protease 2 (SENP2) at a 1500:1 (protein:SENP2) ratio. The proteins were incubated at room temperature for 1 h on an orbital shaker and then dialysed against dialysis buffer overnight at 4°C. The samples were centrifuged at 4000*g* for 30 min to remove protein precipitation. The proteins were concentrated to 3 ml and loaded onto a HiLoad 16/600 Superdex 200 gel-filtration column (GE Healthcare, Chicago, Illinois, USA) pre-equilibrated with 25 m*M* HEPES pH 7.5, 250 m*M* NaCl, 10 m*M* β-ME. Fractions containing proteins were pooled together and mixed with storage buffer [final concentrations of 500 m*M* NaCl, 0.5 m*M* tris(2-carboxyethyl) phosphine (TCEP), 5% glycerol]. The proteins were then flash-frozen in liquid nitrogen and stored at −80°C.

### 2.3. Site-directed mutagenesis   

R264H primers (forward, GATGCGGGTGTCACTGGCGCTAAGATTATTGTGGACACC; reverse, GATGCGGGTGTCACTGGCCATAAGATTATTGTGGACACC) for site-directed mutagenesis were designed using *SnapGene* (GSL Biotech; available at https://www.snapgene.com/). The desired mutated plasmid was obtained using CloneAmp HiFi PCR Premix (Clontech, Mountain View, California, USA) as described previously by Panmanee *et al.* (2019 ▸). The desired mutated plasmid was stored at −20°C.

### 2.4. MATα1–MATβ complex formation   

Complex formation was performed following the protocol described by Murray *et al.* (2014 ▸). Briefly, MATα1 was incubated with MATβV1 or MATβV2 in a 1:2 ratio for 1 h at 4°C in a buffer consisting of 50 m*M* HEPES pH 7.5, 10 m*M* MgCl_2_, 50 m*M* KCl. The complex was then loaded onto a Superdex 200 10/300 gel-filtration column pre-equilibrated with 25 m*M* HEPES pH 7.5 containing 200 m*M* NaCl, 1 m*M* MgCl_2_, 5 m*M* KCl and 1 m*M* TCEP.

### 2.5. Activity assay   

The enzyme-activity assay was performed following the protocol described by Murray *et al.* (2014 ▸). The final concentration of the proteins was 50 n*M* and the final concentration of SCR0911 was 10 µ*M*. The synthesis of SCR0911 has been described by Charoensutthivarakul *et al.* (2015 ▸). The SAMe synthetic activity was measured by measuring the production of SAMe. The reactions were stopped by adding 50 µl 100 m*M* EDTA. SAMe formation was analysed using an *S*-adenosyl­methionine enzyme-linked immunosorbent assay (ELISA) kit (Cell Biolabs, San Diego, California, USA) following the manufacturer’s protocol. The measurements were performed in triplicate. Data were presented as the mean ± the standard error of the mean (SEM). Significance was assessed using a one-way analysis of variance (ANOVA) followed by Tukey–Kramer tests using *GraphPad Prism* version 5. *P* values of less than 0.05 were considered to be significant.

### 2.6. Crystallization and data collection   

The R264H mutant protein was concentrated to 5 mg ml^−1^ and pre-equilibrated with substrates [10 m*M* methionine and 150 µ*M* AMP-PNP (adenylyl-imidodiphosphate), a non­hydrolysable analogue of ATP] in 50 m*M* HEPES buffer pH 7.5 containing 10 m*M* MgCl_2_, 50 m*M* KCl and 10 m*M* dithiothreitol (DTT) for 30 min before crystallization. The wild-type protein was concentrated to 5 mg ml^−1^ and pre-equilibrated with 50 m*M* HEPES buffer pH 7.5 containing 10 m*M* MgCl_2_, 50 m*M* KCl and 10 m*M* DTT before crystallization. Crystal drops consisting of 1 µl protein solution and 1 µl reservoir solution (200 m*M* NaF, 20% PEG 3350, 15% ethylene glycol pH 8.0) were equilibrated against reservoir solution. Crystals appeared at 25°C within three days. Prior to data collection, crystals were flash-cooled in reservoir solution with an additional 20% ethylene glycol. Data were collected at Diamond Light Source, Oxford, England. Data for the R264H MATα1 crystal were collected on beamline I03 at a wavelength of 0.9762 Å using an EIGER2 XE 16M detector. The data for wild-type MATα1 were collected on beamline I04 at a wavelength of 0.9795 Å using an EIGER2 XE 16M detector. Data were integrated using *iMosflm* (Battye *et al.*, 2011 ▸) and scaled using *AIMLESS* (Evans & Murshudov, 2013 ▸) as implemented in the *CCP*4*i* interface (Winn *et al.*, 2011 ▸). The crystal structures were solved by *MOLREP* (Vagin & Teplyakov, 2010 ▸) using human MATα1 (PDB entry 2obv; Shafqat *et al.*, 2013 ▸) as a search model. Model building and restrained refinement were carried out using *Coot* (Emsley *et al.*, 2010 ▸) and *REFMAC*5 (Murshudov *et al.*, 2011 ▸). For the lower resolution R264H mutant structure, noncrystallographic symmetry (NCS) restraints were used during refinement. Crystallo­graphic data-collection and refinement statistics are given in Table 1 ▸.

### 2.7. Differential scanning fluorimetry (DSF)   

DSF was performed as described previously by Panmanee *et al.* (2019 ▸). Each well (20 µl) consisted of 10 µl 0.5 mg ml^−1^ wild-type MATα1 or R264H mutant (dimer or tetramer) in 10 m*M* HEPES buffer pH 7.5 containing 500 m*M* NaCl, 5%(*v*/*v*) glycerol, 0.5 m*M* TCEP and 10 µl 10× SYPRO Orange protein gel stain (Life Technologies, Carlsbad, California, USA). Data were analysed using the *MATLAB* executable *TmTool* following the *TmTool* Quick Set-Up Guide (Life Technologies, Carlsbad, California, USA). Data were represented as the mean *T*_m_ of the three independent experiments ± the SEM.

### 2.8. Molecular docking   

Prediction of the SCR0911 binding site was carried out following the method described in Panmanee *et al.* (2019 ▸). Briefly, blind docking was performed using *SwissDock* (Grosdidier *et al.*, 2011*a* ▸,*b* ▸). Numerous binding modes were obtained in the vicinity of all target cavities (blind docking). A Tripos Mol2 file (.mol2) for SCR0911 was created using a *MarvinSketch* tool (https://chemaxon.com/products/marvin). The crystal structure of the R264H mutant excluding all ligands was used as the target model. The target protein model in PDB file format and ligand in Mol2 format were uploaded using the web-browser interface (http://www.swissdock.ch/docking). Once the docking processes had completed, all possible binding clusters could be downloaded and visualized using the *ViewDock* tool in the *UCSF Chimera* suite (Pettersen *et al.*, 2004 ▸). The best binding pose was selected based on the lowest Gibbs free energy (Δ*G*) and FullFitness score. The protein–ligand complex model was written out in PDB file format using a *UCSF Chimera* tool. The protein–ligand interaction profiles were generated and visualized using *DS Visualizer* (Dassault Systèmes BIOVIA, San Diego, California, USA).

### 2.9. MAT sequence analysis   

Multiple sequence-alignment analysis of the human MATα1 protein was performed with 2963 amino-acid sequences from the MAT protein family using *GREMLIN* conservation analysis (Ovchinnikov *et al.*, 2014 ▸). Sequence-conservation analysis was performed using *WebLogo* 3 (Crooks *et al.*, 2004 ▸).

## 3. Results and discussion   

### 3.1. The crystal structure of tetrameric wild-type MATα1: an apo form   

**Figure 1:**
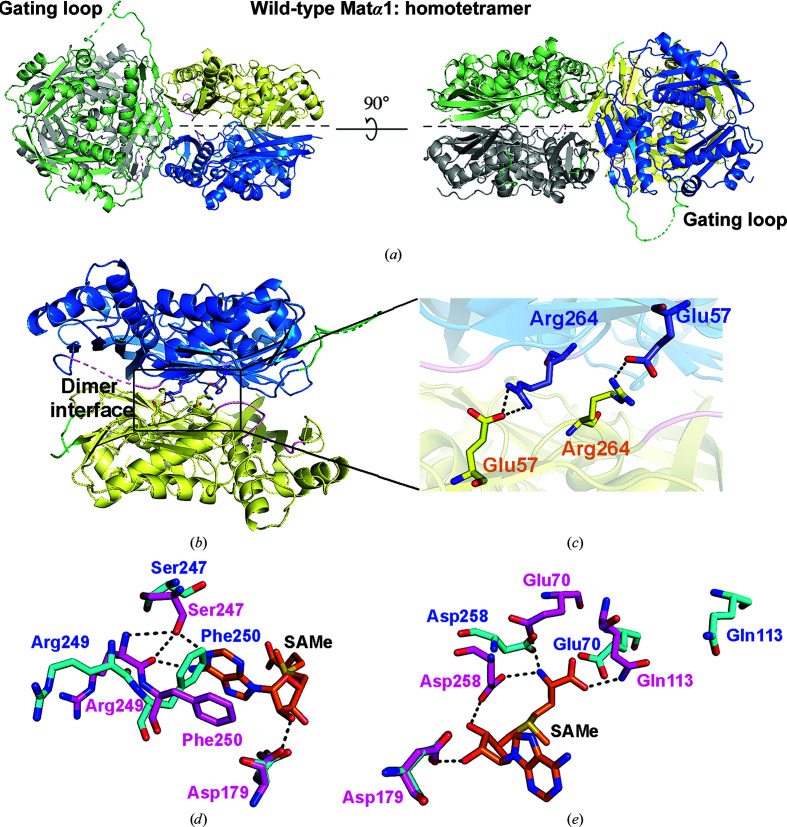
The structure of apo wild-type MATα1. (*a*) The subunits of the wild-type MATα1 tetramer are shown in grey, green, yellow and blue. The tetramer is composed of two self-assembled dimers (four subunits of wild-type MATα1). (*b*) A homodimer is shown as blue and yellow ribbons. The gating loop (113–131) and residues 250–259 are shown in green and pink, respectively. The central part of the dimer interface is marked by a black square. Arg264 and Glu57 of each partner subunit form a salt bridge and stabilize dimer formation. (*c*) The interaction of Arg264 and Glu57 at the dimer interface is illustrated. Hydrogen bonds are represented by black dotted lines. The Arg264 and Glu57 residues of each subunit are labelled in different colours (blue and orange). (*d*, *e*) The active residues that interact with SAMe in the apo and SAMe-bound structures are shown as blue and pink sticks, respectively. The SAMe-bound structure was obtained from the Protein Data Bank (PDB entry 2obv). Hydrogen bonds are represented by black dotted lines.

We report the first crystal structure of human apo MATα1, which was solved at 2.3 Å resolution, revealing a tetrameric assembly. The crystal belonged to space group *C*2, with 45% solvent content. The asymmetric unit contains two dimers comprised of four subunits (Fig. 1 ▸ *a*). Two equivalent active sites are present per dimer, composed of residues from both subunits. Each MATα1 subunit consists of 395 residues, within which there are three major regions that are invisible in the electron-density maps of all subunits (chains *A*–*D*). The first region is the N-terminal region Met1–Glu15, which has been found to be missing in most reported MAT structures owing to its flexibility. The second region is the gating-loop region, Asp116–Glu127, which has also been found to be flexible in the *E. coli* MAT (*e*MAT), rat liver MAT (*r*MAT) and human MATα1/MATα2 structures. This loop has been suggested to regulate access to the active site by adopting an open or closed conformation. The SAMe-bound structure of MATα1 was found to have a closed conformation of the gating loop, with the binding of SAMe being partially stabilized by this loop (Shafqat *et al.*, 2013 ▸). The gating loop is generally in the open conformation and is not present in the unliganded structure (apo form). The open conformation of the gating loop allows the active site to be solvent-accessible (Shafqat *et al.*, 2013 ▸). The last invisible region is the Phe250–Ala259 domain, which contains methionine-binding sites located at the dimer interface. Flexibility of this region has been reported in the *r*MAT structure (González *et al.*, 2000 ▸), but it was clearly seen in the apo structure of human MATα2 despite the similarity of its sequence to that of MATα1 (Panmanee *et al.*, 2019 ▸). When substrates bind to the active site, the gating loop adopts the closed conformation owing to the stabilization of the Phe250–Ala259 region by the presence of SAMe (Komoto *et al.*, 2004 ▸; Murray *et al.*, 2014 ▸, 2016 ▸; Shafqat *et al.*, 2013 ▸). In addition, the Asp94–Phe99 domain is absent in chains *A* and *D* of the present apo MATα1 structure. The average temperature factors (*B* factors) of this region calculated for chains *B* and *C* were 82.0 and 82.2 Å^2^, respectively, while overall *B* factors of 33.8 and 38.6 Å^2^ were found for chains *B* and *C*. The higher *B* factor for this domain (Asp94–Phe99) compared with the average *B* factor of the entire chain indicates that this domain is very flexible; it thus becomes disordered and eventually becomes invisible in chains *A* and *D*. This region is located on the solvent-exposed surface and features as a turning-loop domain connecting the α-helix (Tyr79–Ile90) and β-sheet (Lys53–Thr72 and Asn105–Gln112) domains.

Each subunit of the dimer largely interacts via β-sheets in an inverse contact. This generates two active sites per dimer at the dimer interface (Fig. 1 ▸ *b*). Only a few polar interactions are found at the dimer interface, mainly involving the formation of salt bridges between Arg264 and its partner Glu57 from the interacting subunit (Fig. 1 ▸ *c*).

The previously reported SAMe-bound structure (PDB entry 2obv) contains one MATα1 monomer in the asymmetric unit in space group *I*222 (Shafqat *et al.*, 2013 ▸). The biological assemblies of an identical subunit in a dimer and a tetramer were generated by twofold-symmetry axes. Structural comparison of the active-site residues of the SAMe-bound structure and the present apo structure of MATα1 revealed an overall root-mean-square deviation (r.m.s.d.) of 0.687 Å. These two structures align well, except for the active site and the gating-loop regions that adopt the conformations observed in the SAMe-bound structure. At the active site, the aromatic ring of Phe250 in the apo structure is located in the SAMe-binding site, where it forms π–π stacking with the adenine moiety of SAMe in the SAMe-bound structure (Fig. 1 ▸ *d*; Shafqat *et al.*, 2013 ▸). In addition, the O atom of Ser247, the OE1 atom of Glu70 and the OD1 atom of Asp258 of the apo structure move by 3.9, 6.5 and 3.9 Å, respectively, from their positions in the SAMe-bound structure (Figs. 1 ▸ *d* and 1 ▸ *e*). These three residues (Ser247, Glu70 and Asp258) have been reported to interact with methionine and ATP during SAMe formation (Murray *et al.*, 2016 ▸; Shafqat *et al.*, 2013 ▸). The active-site residues that interact with the substrates methionine and ATP are contributed by both of the dimeric subunits. The main chain of methionine interacts with Glu70, Gln113 and Asp258, while the positioning of ATP in the active site is stabilized by Asp179, Ser247, Arg249 and Phe250. According to the conformational change of the gating loop upon substrate binding and SAMe production, Gln113, which directly binds to methionine and SAMe and is also a part of the gating loop, shows a movement of 8.1 Å in the present apo structure (Fig. 1 ▸ *e*).

### 3.2. The crystal structure of the dimeric MATα1 R264H mutant   

**Figure 2:**
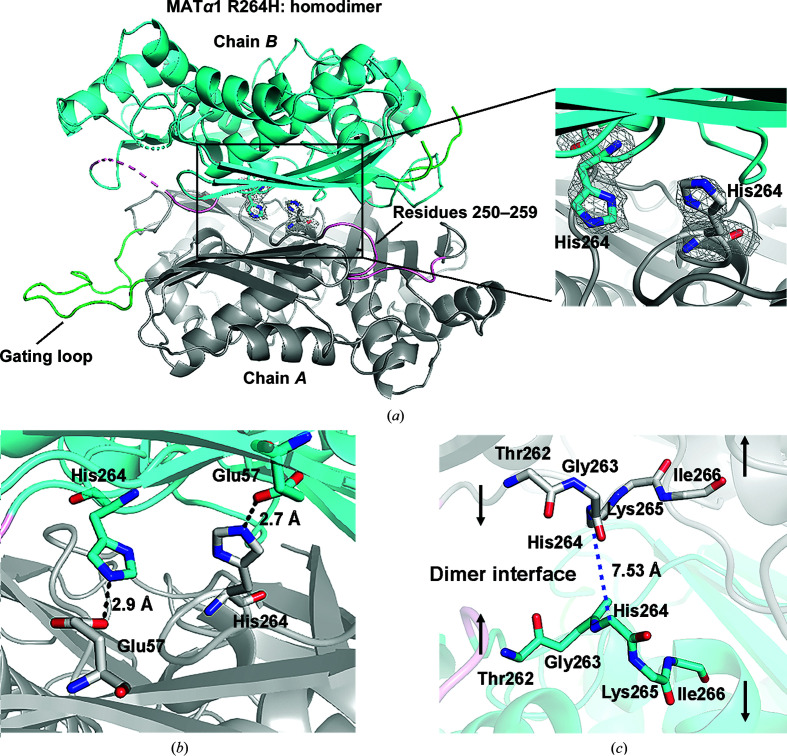
The structure of the apo MATα1 R264H mutant. (*a*) The subunits of the MATα1 R264H dimer are shown as grey and blue ribbons. The dimer is composed of two self-assemblies of the R264H subunit (chains *A* and *B*). The Arg264His mutation site is shown in the *F*_o_ − *F*_c_ OMIT map contoured at the 3σ level (grey). The gating loop (113–131) and residues 250–259 are shown in green and pink, respectively. (*b*) His264 and Glu57 of each partner subunit form hydrogen bonds of 2.7 and 2.9 Å, aiding stabilization of the dimer. Hydrogen bonds are shown as black dotted lines. (*c*) The dimer interface of the MATα1 R264H mutant is shown. The substitution of Arg264 by His264 changes the distance between this residue of each subunit to 7.53 Å (C^α^–C^α^ measurement), which is about 2.06 Å longer than the corresponding distance in the wild type. The Thr262–Ile266 main chains are shown as grey and blue sticks.

We have determined the crystal structure of the MATα1 R264H mutant at 2.85 Å resolution. The crystal belonged to space group *C*2, with 42% solvent content. The asymmetric unit contains a dimer created by two subunits (chains *A* and *B*), with the R264H mutation sites clearly visible in the *F*_o_ − *F*_c_ OMIT map (Fig. 2 ▸ *a*). Chain *B* contains three invisible regions, the N-terminus (Met1–Glu15), the gating loop (Asp116–Glu127) and the Phe250–Gly257 region, while chain *A* contains an ordered gating loop and the Phe250–Gly257 region shows a clear electron-density map. These two regions (Phe250–Gly257 and the gating loop) are involved in stabilizing substrate binding, so they become more flexible in the apo form. Chain *A* shows an overall *B* factor of 60.74 Å^2^, which is slightly lower than the overall *B* factor of chain *B* (61.72 Å^2^). Chains *A* and *B* align well, with an r.m.s.d. of 0.076 Å, and are almost identical, despite some differences in regional flexibility.

The Arg265His mutant of *r*MAT and the corresponding R264H mutant of human MATα1 were reported to be unable to dimerize (Chamberlin *et al.*, 1997 ▸; Pérez Mato *et al.*, 2001 ▸), whereas the analogous *e*MAT Arg244His mutant remained as a tetramer (Reczkowski *et al.*, 1998 ▸). We have previously reported that the R264H mutant of MATα1 is found in oligomeric states (Panmanee *et al.*, 2019 ▸); however, we were unable to produce a good diffraction-quality crystal of this mutant. Thus, we undertook a structural study of the Arg264Ala (R264A) mutant of MATα2 in order to understand the role of Arg264 in MAT catalysis. We determined its structure at 1.7 Å resolution and found that Arg264 is involved in interaction with tripolyphosphate during enzyme catalysis, while the dimer interfaces of the R264A mutant and the wild-type MATα2 structure were in a similar orientation and no significant movement was observed (Panmanee *et al.*, 2019 ▸).

**Figure 3:**
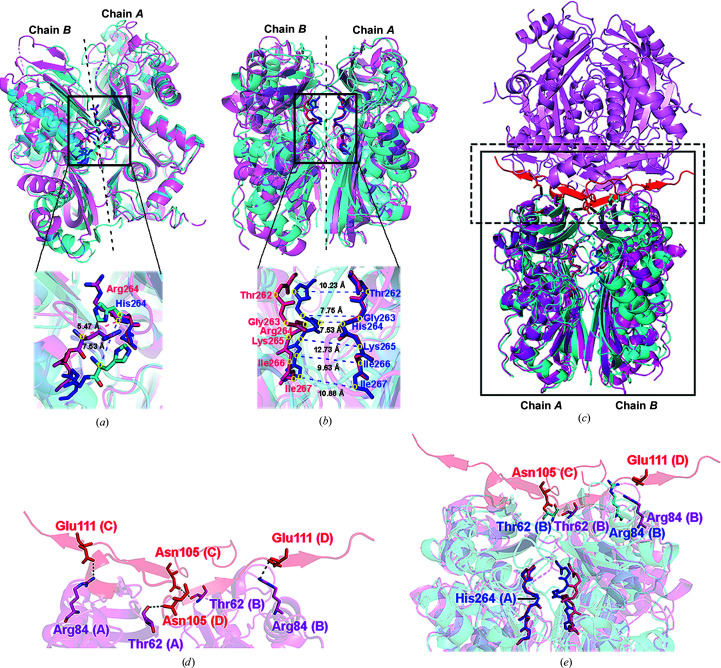
Structural comparison of the apo form of wild-type MATα1 and the R264H mutant. Wild-type MATα1 and the MATα1 R264H mutant are shown as pink and blue ribbons, respectively, with alignment of chain *A* of each protein. (*a*) The dimer interface is represented by dashed lines; the C^α^–C^α^ distances between the Arg264 or His264 residues of each protein are illustrated. C^α^ atoms are marked by yellow circles. (*b*) The distance displacements caused by the R264H mutation are shown by C^α^–C^α^ measurements (blue dots) of these residues (Val262–Ile267) in each subunit compared with the wild type. The Thr262–Ile266 main chains are shown as dark pink (wild type) and blue (R264H mutant) sticks. C^α^ atoms are marked by yellow circles. (*c*) The dimer–dimer interface of wild-type MATα1 is shown in red (chains *C* and *D*). The areas in the dashed and solid boxes are enlarged in (*d*) and (*e*), respectively. (*d*) The polar interactions at the dimer–dimer interface found in the wild type are illustrated for chain *A* with chain *C* and for chain *B* with chain *D*. The residues of chains *A* and *B* are shown as pink sticks (pink labels), while the residues of chains *C* and *D* are shown as red sticks (red labels). (*e*) The distance displacements of Thr62 and Arg84 caused by the R264H mutation are shown compared with the wild type. Thr62 and Arg84 are presented as pink and red sticks for the wild type and as blue sticks for the R264H mutant. The Thr262–Ile266 main chains are shown as dark pink (wild type) and blue (R264H mutant) sticks to mark the mutation regions.

In contrast, the crystal structure of the R264H mutant of MATα1 described in the present study shows a huge difference in the dimer-interface orientation owing to the substitution of Arg264 in the central part of the dimer by His. His264 forms stronger hydrogen bonds to Glu57 (Fig. 2 ▸ *b*) than those found in the wild type (Arg264), as the shorter side chain of histidine causes the displacement of the residues located at the dimer interface (Fig. 3 ▸). At the mutation site, the distance between His264 in each subunit is 7.53 Å in the R264H mutant, which is around 2.06 Å longer compared with the distance between the Arg264 residues in the wild type (5.47 Å) (Figs. 2 ▸ *c* and 3 ▸ *a*). The residues that line the dimer interface change their orientation in order to accommodate the mutation, causing the interaction at one part (Thr262–Gly263) of the dimer interface to tighten in comparison to the other part (Arg264–Ile267) (Fig. 3 ▸ *b*). The upper part of the mutation site becomes generally tighter (Thr262–Gly263) along the dimer interface, while the mutation site itself loosens. The displacements caused by the R264H mutation compared with wild-type MATα1 are shown in Table 2 ▸. The dimer interface was examined using *PDBePISA* to investigate the additional alterations derived from this mutation (Krissinel & Henrick, 2007 ▸). This analysis reveals that the solvent-accessible surface area of the R264H assembly is 52 110 Å^2^, which is consistent with the value of 52 100 Å^2^ found for the wild type. However, the numbers of residues that are involved in polar interactions at the dimer interface differ. The dimer interface of R264H is associated with 25 hydrogen bonds and nine salt-bridge interactions, while only 18 hydrogen bonds and four salt bridges are observed at the wild-type dimer interface (Supplementary Table S1). In addition, Δ*G* ^diss^, which corresponds to the free-energy difference between the associated and dissociated states, is 7.1 and 3.1 kcal mol^−1^ for the R264H mutant and wild-type assemblies, respectively. These alterations at the dimer interface of the R264H mutant assembly indicate that His264 generates amino-acid displacement along the dimer interface.

**Figure 4:**
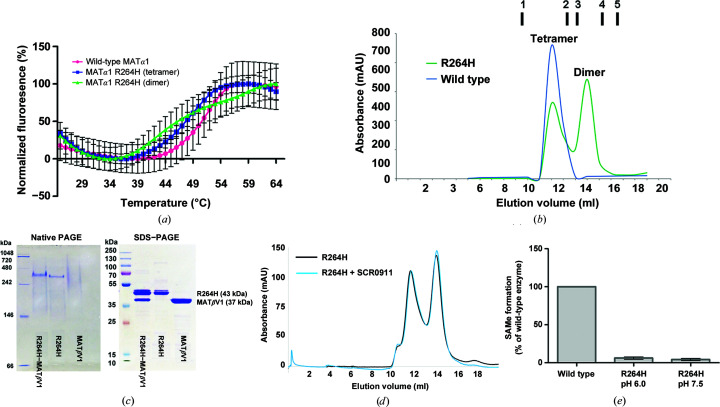
The characteristics of the R264H mutant in solution. (*a*) A thermal shift assay shows the *T*_m_ of the R264H mutant (dimer and tetramer) compared with the wild type. (*b*) Gel-filtration profiles of wild-type MATα1 (blue) and the R264H mutant (green) are shown by the absorbance at *A*_280_. The vertical markers (1–5) represent the elution volumes of standard proteins (GE Healthcare): ferritin (1; 440 kDa), aldolase (2; 158 kDa), conalbumin (3; 75 kDa), ovalbumin (4; 43 kDa) and ribonuclease (5; 13.7 kDa). (*c*) Native PAGE and SDS–PAGE gels of the MATα1 R264H–MATβV1 fraction are presented with those of R264H MATα1 and MATβV1 on their own. (*d*) Gel-filtration profiles of the R264H mutant with or without SCR0911 incubation are shown. (*e*) SAMe production by the R264H mutant at pH 6.0 and 7.5 compared with the wild type. Data are the mean ± SEM (*n* = 3).

Pérez Mato *et al.* (2001 ▸) demonstrated that the mutation of Arg265 in the rat enzyme (equivalent to Arg264 in the human enzyme) to His (R265H) resulted in a monomeric MAT which catalysed only 0.37% of the SAMe production of the wild type. To mimic the dominant inherited form of the R264H mutant that causes hypermethioninemia, wild-type and R265H subunits of the rat enzyme were mixed and assembled as an R265H–wt hetero-oligomer. It was found that the R265H mutant could oligomerize with the wild-type subunit as a heterodimer (R265H–wt MAT; Mato *et al.*, 2001 ▸). However, the R265H–wt dimer was unable to synthesize SAMe, suggesting its dominant effect, as was found in patients with IPH. Additionally, the tripolyphosphatase activity was found to be comparable to that of the wild type in the hybrid MAT, but could not be stimulated by methionine and ATP, indicating impairment of substrate binding (Mato *et al.*, 2001 ▸). The thermal stability of the R264H mutant is lower than that of the wild-type enzyme, with *T*_m_ values of 47.50 ± 0.18 and 47.27 ± 0.37°C for the tetramer and the dimer, respectively, compared with 50.3 ± 0.10°C for the wild-type enzyme (Fig. 4 ▸ *a*).

All attempts to obtain a holo structure of the MATα1 R264H mutant failed and produced crystal structures of the apo form, despite the R264H mutant protein being mixed with substrates (methionine and AMP-PNP) prior to crystallization. The methionine substrate concentration giving a half-maximum of the reaction rate (*K*_m_) of tetrameric MATα1 has been reported to be ∼23 µ*M* (Cai *et al.*, 1996 ▸; Lombardini & Sufrin, 1983 ▸). A methionine concentration of 200 µ*M*, which is approximately tenfold greater than the *K*_m_ of the wild type, was used in our SAMe synthetic assays for the R264H mutant and a loss of SAMe production was still observed, suggesting a lower affinity of the enzyme–substrate complex. The loss of enzymatic activity caused by this mutation may thus originate from its inability to bind the substrates in the first place, resulting from the change in the positions of the residues at the dimer interface, which is further compromised by causing a lower affinity for a tetrameric assembly and a greater propensity for dimeric assembly.

### 3.3. The R264H mutation weakens the dimer–dimer interface in MATα1 and reduces the tetramer affinity   

The subunit interactions between and within the dimer play a crucial role in forming the tetramer, as shown in Figs. 3 ▸(*c*)–3 ▸(*e*). Our data show that the R264H mutant exists as a dimer and a tetramer in solution in almost equal proportions, in contrast to the wild-type enzyme, which exists primarily as a tetramer (Fig. 4 ▸ *b*). The tetrameric form of the wild-type MATα1 structure reveals a dimer–dimer contact surface which involves a few polar interactions, including those between Thr62 (chains *A* and *B*) and Asn105 (chains *C* and *D*), and between Arg84 (chains *A* and *B*) and Glu111 (chains *C* and *D*) (Fig. 3 ▸ *d*). Also, no disulfide bonds are observed in either the dimer or tetramer subunit interactions. The R264H mutation causes the residues at the dimer interface to be displaced from their original positions in the wild type. Accordingly, the overall protein architectures are changed despite the preservation of the secondary structure. In the mutant, Thr62 and Arg84, which participate in dimer–dimer contact interactions, are relocated by 8.6 and 9.8 Å, respectively, compared with the wild-type structure (Fig. 3 ▸ *e*), resulting in a reduced tetramer affinity and the ability to form a tetrameric assembly of the mutant enzyme.

### 3.4. Can the loss of activity of R264H MATα1 be recovered?   

**Figure 5:**
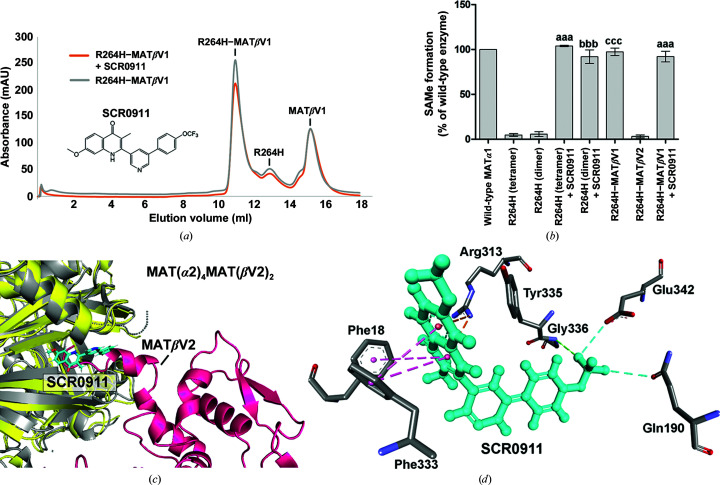
The effect of regulatory subunits (MATβ) and SCR0911 on the activity of MATα1 R264H. (*a*) Gel-filtration profiles of MATα1 R264H–MATβV1 with or without SCR0911 pre-incubation are shown by the absorbance at *A*_280_. (*b*) The effects of the regulatory subunits (MATβV1/V2) and SCR0911 on the enzymatic activity of MATα1 R264H are shown by measuring SAMe formation. ‘aaa’, ‘bbb’ and ‘ccc’ denote statistical significance at *p* < 0.001 compared with tetrametric R264H, dimeric R264H (dimer) and both oligomeric forms of R264H, respectively. Data are the mean ± SEM (*n* = 3). (*c*) The SCR0911 binding site of the R264H mutant (grey) obtained by molecular docking is aligned with the structure of the complex of MATα2 (yellow) and MATβ (pink). SCR0911 is shown as blue sticks. (*d*) The SCR0911 and R264H interaction profiles obtained from *SwissDock* are illustrated. Protein–ligand interactions are shown in black for hydrogen bonds, blue for halogen bonds, pink for π–π stacking and orange for electrostatic interactions.

MATα1 is a liver-specific enzyme and is found to act as a homotetramer or a homodimer, while MATα2 functions as a hetero-oligomer by forming complexes, MAT(α2)_4_(βV1)_2_ or MAT(α2)_4_(βV2)_2_, with its regulatory subunits MATβV1 or MATβV2, respectively (Murray *et al.*, 2014 ▸). The hetero-complex structure of MATα2 revealed that two subunits of MATβ interact with the tetrameric form of MATα2 at the dimer interface. MATβ has two isoforms, MATβV1 and MATβV2, that differ in the length and the identity of the first 20 amino acids at the N-terminus but exhibit total conservation in the rest of the primary structure (Yang *et al.*, 2008 ▸). An *in vitro* study suggested that MATα1 could also form a hetero-complex with MATβV1 and to a lesser extent with MATβV2 (Murray *et al.*, 2014 ▸). Here, we found that only MATβV1 was able to form a hetero-complex with the R264H MATα1 mutant (Figs. 4 ▸ *c* and 5 ▸ *a*), providing a recovery of the enzymatic activity (SAMe production) of the mutant to the level of wild-type MATα1 (Fig. 5 ▸ *b*). We have previously reported a series of quinolone-based compounds that could regulate the activities of MAT enzymes (Panmanee *et al.*, 2019 ▸). We tested the effect of the compound SCR0911 on the MATα1 R264H mutant and discovered that it could also recover the activity of the mutant to the same level as wild-type MATα1 (Fig. 5 ▸ *b*). However, pre-incubation with SCR0911 did not prevent hetero-complex formation of the R264H mutant and MATβV1, suggesting that MATβV1 is a competitive cognate partner of the R264H mutant. Also, the addition of SCR0911 to the MATα1 R264H–MATβV1 complex did not give a synergistic effect, suggesting that SCR0911 competes with MATβV1 for binding at the same dimeric interface and that MATβV1 binding prevents SCR0911 from interacting with the mutant protein (Figs. 5 ▸ *a* and 5 ▸ *b*).

MATβV1 was able to alter the activity of MATα2 without changing its catalytic transition state (Firestone & Schramm, 2017 ▸; Murray *et al.*, 2014 ▸), and the catalytic site of MATα2 was also preserved in the presence of MATβ (Murray *et al.*, 2016 ▸), indicating that the increase in activity is modulated by allo­steric regulation. The restoration of the activity of the MATα1 R264H mutant by the regulatory subunit MATβV1 or the compound SCR0911 suggests a similar binding interface. The best binding position of SCR0911 in the R264H mutant was predicted by blind docking using *SwissDock* (FullFitness score of −3475.04 kcal mol^−1^ and Δ*G* of −7.76 kcal mol^−1^). The protein–ligand interaction profiles are shown in Figs. 5 ▸(*c*) and 5 ▸(*d*). The predicted site is in the same dimeric interface pocket where MATβ and the compound PF-9366 have been reported to bind (Murray *et al.*, 2014 ▸; Quinlan *et al.*, 2017 ▸). We also used alternative software (*PatchDock*) to calculate the best binding position of SCR0911 in the R264H mutant (Duhovny *et al.*, 2002 ▸; Schneidman-Duhovny *et al.*, 2003 ▸). The protein–ligand interaction site predicted by *PatchDock* is at a similar position to that predicted by *SwissDock* (Supplementary Fig. S1). Among the predicted ligand-interacting residues, Arg313 and Tyr335 are known to interact with MATβ subunits (Murray *et al.*, 2014 ▸). Efforts to obtain a crystallographic structure containting the compound were not successful, suggesting some flexibility, heterogeneity and/or weaker binding compared with MATβ, which is consistent with the inability of SCR0911 to compete with MATβ in restoring the SAMe-production activity of the MATα1 R264H mutant.

Neither the tetramer nor the dimer fraction from the gel-filtration column could catalyse the production of SAMe (Fig. 5 ▸ *b*). Therefore, we presume that the impaired function of the R264H mutant is caused by distortion of the active site rather than impaired tetramerization. To test this hypothesis, 100 µ*M* SCR0911 was incubated with R264H mutant protein for 2 h at 4°C prior to performing size-exclusion chromatography. The result shows that the compound does not change the dimer–tetramer equilibrium of the R264H mutant (Fig. 4 ▸ *d*). In addition to hypermethioninemic conditions, the protonation state of the R264H mutant is changed at physiological pH (about 7.4) owing to the effect of mutation. Arg has a p*K*_a_ of about 12.48, while His has a p*K*_a_ of 6.0, and therefore Arg is fully protonated at physiological pH. Under our experimental conditions the enzyme reactions were performed at pH 7.5, so Arg264 of the wild-type enzyme is fully protonated. To test whether the R264H mutant is able to produce SAMe when the His residue is protonated, the catalytic reaction was performed at pH 6.0. We found that the lower pH could not recover SAMe production compared with the wild-type enzyme (Fig. 4 ▸ *e*), suggesting that the loss of catalytic efficiency is likely to be owing to the disordered active site.

### 3.5. Sequence-conservation analysis and insight from the structural analysis of MAT enzymes   

**Figure 6:**
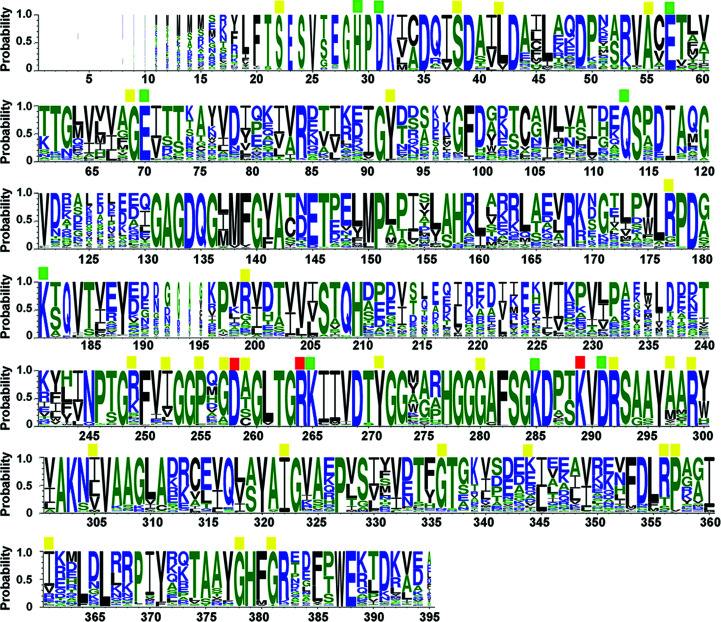
Amino-acid conservation of MATα1 showing catalytic residues and mutation sites causing hypermethioninemia. Different colours are used to show amino-acid hydrophobicity (blue, hydrophilic; green, neutral; black, hydrophobic). Catalytic residues are marked by green squares. Residues reported to have mutations and cause hypermethioninemia are marked with yellow squares. Red squares indicate residues that are involved in both catalysis and missense mutations.

**Figure 7:**
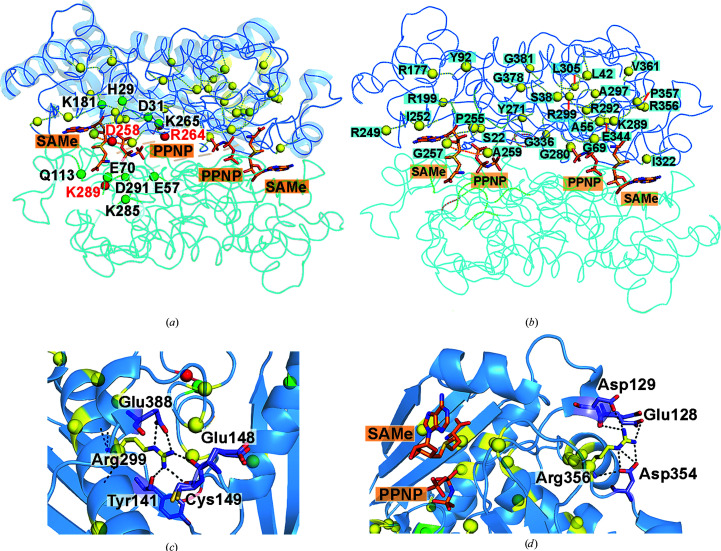
Catalytic residues of MATα1 and mutation sites causing IPH. (*a*) Two active sites of MATα2 are shown with SAMe and PPNP molecules. Residues involved in catalysis are shown as green spheres. Missense mutations causing hypermethioninemia are shown as yellow spheres. Red spheres represent residues that are involved in both catalysis and missense mutations causing hypermethioninemia. (*b*) Missense mutations causing hypermethioninemia are shown as yellow spheres. The two subunits are shown in dark and light blue using the MATα2 structure (PDB entry 5a1i; Murray *et al.*, 2016 ▸) as a model. (*c*, *d*) The Arg299 (*c*) and Arg356 (*d*) interaction sites are illustrated. Black dotted lines indicate hydrogen bonds and the interacting residues are shown as sticks.

Multiple sequence alignments of proteins homologous to MAT from all living organisms were performed using 2963 protein sequences (Fig. 6 ▸). The residues involved in MAT catalysis include five acidic amino acids (Asp31, Glu57, Glu70, Asp258 and Asp291), six basic amino acids (His29, Lys181, Arg264, Lys265, Lys285 and Lys289) and one neutral amino acid (Gln113) (Figs. 6 ▸ and 7 ▸ *a*). All of these catalytic residues are highly conserved throughout evolution (Fig. 6 ▸). Three of these catalytic residues (Asp258, Arg264 and Lys289) play a direct role in substrate binding and have been reported to cause IPH when the *MAT1A* gene encodes mutations at these residues: Asp258Gly, Arg264His/Cys and Lys289Asn (Chamberlin *et al.*, 2000 ▸; Chien *et al.*, 2005 ▸; Fernández-Irigoyen *et al.*, 2010 ▸; Nagao & Oyanagi, 1997 ▸). All mutation points of the *MAT1A* gene causing IPH that have been reported to date are illustrated in Figs. 6 ▸ and 7 ▸(*b*). Most of the *MAT1A* genes in which missense mutations occur that cause IPH encode fully conserved amino-acid residues (Fig. 7 ▸ *a*): Ser22Leu, Ser38Asn, Ala55Asp, Gly69Ser, Tyr92His, Pro255Ser, Tyr271Cys, Gly280Val, Arg292Cys, Arg299His/Cys, Ile322Val/Met, Val361Phe, Gly378Ser and Gly381Arg (Chamberlin *et al.*, 1996 ▸, 2000 ▸; Chien *et al.*, 2005 ▸; Fernández-Irigoyen *et al.*, 2010 ▸; Linnebank *et al.*, 2005 ▸; Sen *et al.*, 2019 ▸; Tada *et al.*, 2004 ▸; Ubagai *et al.*, 1995 ▸).

In addition to the Arg264His mutation, we attempted to establish the functional role of Arg299. Although we were successful in producing an Arg299His mutant, the majority of this mutant protein formed aggregates in solution. Arg299 is located in the middle of the solvent-inaccessible α-helix domain. The side chain of Arg299 forms hydrogen bonds to Tyr141 of an underlying β-sheet domain and also to Glu148, Cys149 and Glu388 of the connecting loop (Fig. 7 ▸ *c*), suggesting its importance in protein folding and overall structural stability. Some of the IPH-causing mutations are found in highly conserved amino-acid residues: Leu42Pro, Arg177Trp, Arg199Cys, Arg249Trp, Ile252Thr, Gly257Arg, Ala259Val, Ala297Asp, Gly336Arg, Arg356Trp/Pro/Gln and Pro357Leu (Chamberlin *et al.*, 1996 ▸; Chien *et al.*, 2005 ▸; Fernández-Irigoyen *et al.*, 2010 ▸; Linnebank *et al.*, 2005 ▸; Sen *et al.*, 2019 ▸; Ubagai *et al.*, 1995 ▸; Nashabat *et al.*, 2018 ▸; Fig. 5 ▸ *a*). We also attempted to obtain purified Arg356Trp mutant, but its purification exhibited problems similar to those faced with the Arg299His mutant. Arg356 is located on the surface of the solvent-accessible α-helix domain and forms several hydrogen bonds to Glu128, Asp129 of the gating loop and Asp354 of the neighbouring connecting loop (Fig. 7 ▸ *d*), suggesting an important role in the overall protein architecture. Only a few IPH-causing mutations have been found to involve poorly conserved amino acids (Leu305Pro, Glu344Ala and Val361Phe; Fig. 6 ▸; Chamberlin *et al.*, 2000 ▸; Nashabat *et al.*, 2018 ▸; Ubagai *et al.*, 1995 ▸). In addition, some of these mutations (Arg356His and Glu344Ala) have been reported to cause increased susceptibility to thoracic aortic aneurysms when mutations occur in *MAT2A* genes, suggesting their importance for enzyme stability and function (Guo *et al.*, 2015 ▸).

In summary, this study provides a structural basis for the lower activity found for the R264H mutation of MATα1 at the dimer interface. This mutation changes the positions of residues that constitute the dimer interface where the active sites are located, resulting in an inability to bind the substrate and causing a loss in activity. The changes at the dimeric interface also give rise to a lower affinity for a tetrameric assembly and a greater propensity for a dimeric assembly for the R264H MATα1 mutant. Arg264 is also involved in providing enzyme stability by forming a salt bridge with Glu57; its mutation to histidine thus causes lower enzyme stability, as observed by the decreased melting temperature of the mutant. We also show that the activity of the R264H mutant can be restored, presumably by reinstating the active site, when the mutant is incubated with the quinolone compound SCR0911 or with its regulatory subunit MATβV1. This observation provides an opportunity for chemical intervention to alleviate this disease-causing defect in catalysis.

## Supplementary Material

PDB reference: human MATα1, wild type, 6sw5

PDB reference: R264H mutant, 6sw6

Supplementary Figure S1 and Table S1. DOI: 10.1107/S2059798320006002/qh5064sup1.pdf

## Figures and Tables

**Table 1:** Data-collection and refinement statistics Values in parentheses are for the highest resolution shell.

	Wild-type MATα1	R264H MATα1
Data collection		
Space group	*C*2	*C*2
*a*, *b*, *c* (Å)	218.68, 61.00, 119.76	108.56, 83.62, 87.56
α, β, γ (°)	90.00, 90.55, 90.00	90.00, 107.99, 90.00
Resolution (Å)	58.76–2.35 (2.41–2.35)	51.62–2.85 (3.00–2.85)
No. of reflections	66266	17484
*R*_merge_ (%)	10.8 (50.2)	17.7 (85.8)
〈*I*/σ(*I*)〉	6.4 (1.9)	5.1 (1.5)
CC_1/2_	0.991 (0.762)	0.974 (0.755)
Completeness (%)	100.0 (99.9)	99.3 (97.9)
Multiplicity	3.4 (3.4)	5.1 (5.2)
Wilson *B* factor (Å^2^)	29.34	55.57
Refinement		
*R*_work_/*R*_free_ (%)	18.82/23.88	26.07/28.79
No. of atoms		
Protein	11209	5693
Ligand/ion	62	—
Water	408	4
Average *B* factor (Å^2^)		
Protein	36.80	61.23
Ligand	46.88	—
Water	31.89	34.44
R.m.s. deviations		
Bond lengths (Å)	0.0103	0.0071
Bond angles (°)	1.4265	1.1520
PDB code	6sw5	6sw6

**Table 2:** C^α^–C^α^ distances of the residues (Val262–Ile267) in the mutation site of each subunit in comparison to the wild type

	C^α^–C^α^ (Å)	
Residue	Wild type	R264H mutant
Thr262	12.01	10.23
Gly263	10.14	7.75
Arg264/His264	5.47	7.53
Lys265	11.37	12.73
Ile266	9.22	9.63
Ile267	10.58	10.88
