# Supplementary material for: Structural basis of the dominant inheritance of hypermethioninemia associated with the Arg264His mutation in the MAT1A gene
Source: Acta Crystallogr D Struct Biol. 2020 May 29;76(Pt 6):594–607. doi: 10.1107/S2059798320006002 (PMC7271947; doi:10.1107/S2059798320006002)
Supplement: Supplementary file 1 [file d-76-00594-sup1.pdf]

STRUCTURAL BIOLOGY

Volume 76 (2020)

Supporting information for article:

Structural basis of the dominant inheritance of hypermethioninemia associated with the Arg264His mutation in the MAT1A gene

Jiraporn Panmanee, Svetlana V. Antonyuk and S. Samar Hasnain

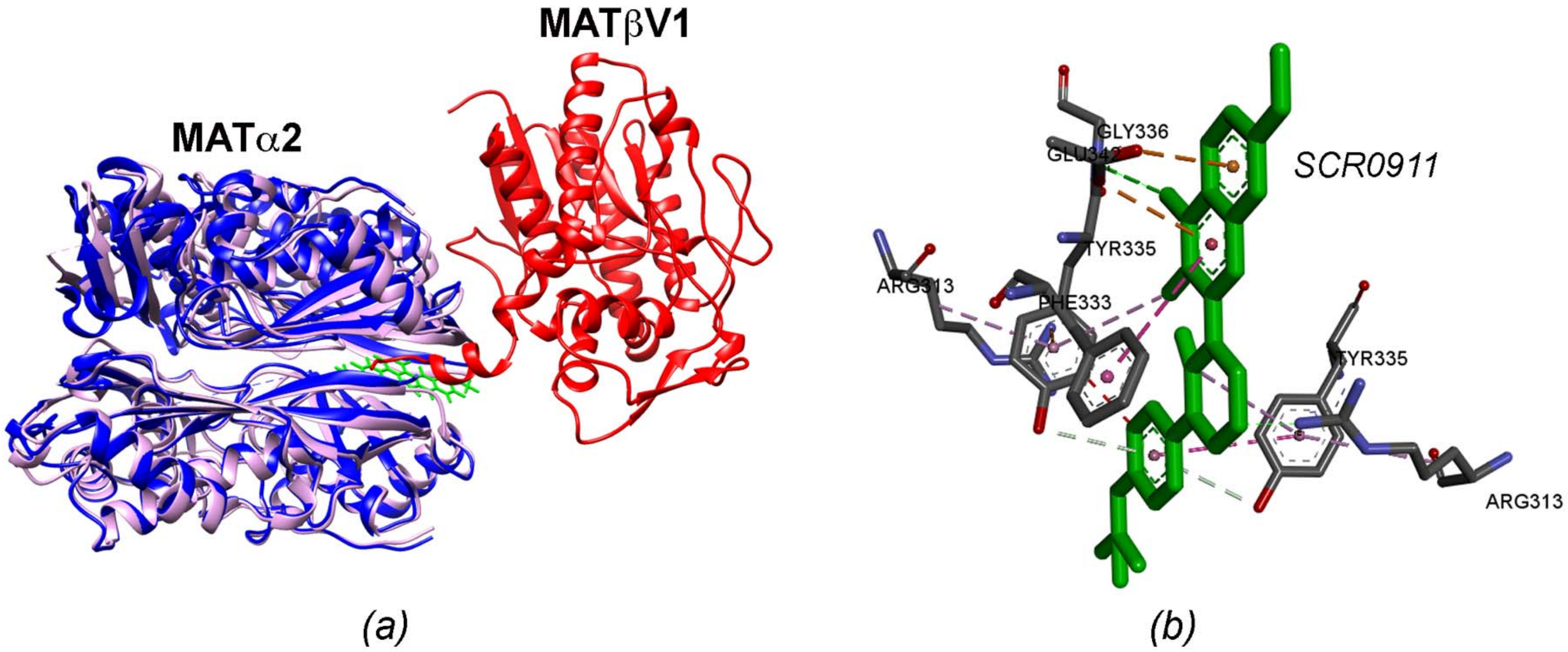

Figure S1 Molecular docking of SCR0911 binding site of the R264H mutant by PacthDock. **(a)** The R264H mutant (grey) is aligned with the complex structure of MATα2 (blue) and MATβ (red). SCR0911 is shown in a green stick. **(b)** SCR0911 and R264H interaction profiles obtained from PacthDock are shown. Protein ligand interactions are represented by green (H-bonds), pink (π-π stacking) and orange colour (electrostatic interactions).

Table S1 Dimer interface analysis showing residues involves in polar interactions.

**wtMATα1**				**R264H**			
Hydrogen bonds				Hydrogen bonds			
#	Chain A	Distance [Å]	Chain B	#	Chain A	Distance [Å]	Chain B
1	B:SER 329[OG]	3.39	A:MET 19[O]	1	B:THR 21[N]	3.24	A:GLN 317[OE1]
2	B:SER 319[OG]	3.03	A:MET 19[O]	2	B:THR 21[OG1]	3.19	A:SER 319[OG]
3	B:ARG 264[NH2]	3.34	A:GLU 57[OE1]	3	B:THR 21[OG1]	3.17	A:SER 329[OG]
4	B:GLN 183[NE2]	2.94	A:GLN 135[O]	4	B:LYS 61[NZ]	3.66	A:GLU 70[OE2]
5	B:ARG 264[NH2]	3.17	A:THR 262[O]	5	B:LEU 137[N]	3.84	A:GLU 23[OE2]
6	B:MET 19[N]	2.99	A:SER 329[OG]	6	B:LYS 181[NZ]	2.95	A:ASP 134[OD1]
7	B:GLN 190[NE2]	3.20	A:GLU 342[OE1]	7	B:LYS 181[NZ]	2.75	A:ASP 134[OD2]
8	B:MET 19[O]	3.36	A:GLN 317[NE2]	8	B:GLN 183[NE2]	2.76	A:TYR 320[O]
9	B:MET 19[O]	3.27	A:SER 319[OG]	9	B:ALA 259[N]	2.76	A:GLU 70[OE2]
10	B:MET 19[O]	3.49	A:SER 329[OG]	10	B:HIS 264[NE2]	2.97	A:GLU 57[OE1]
11	B:GLU 57[OE1]	3.19	A:ALA 259[N]	11	B:GLN 317[NE2]	2.96	A:THR 21[O]
12	B:GLU 57[OE2]	2.47	A:ARG 264[NH2]	12	B:SER 319[OG]	3.15	A:THR 21[OG1]
13	B:GLN 135[OE1]	2.41	A:THR 185[OG1]	13	B:SER 329[OG]	3.19	A:THR 21[OG1]
14	B:GLN 135[OE1]	3.26	A:THR 21[OG1]	14	B:SER 331[OG]	2.59	A:MET 19[O]
15	B:THR 185[OG1]	2.79	A:GLN 135[NE2]	15	B:GLN 317[OE1]	3.32	A:THR 21[N]
16	B:THR 262[O]	2.92	A:ARG 264[NH2]	16	B:GLU 70[OE2]	3.88	A:LYS 61[NZ]
17	B:SER 329[OG]	2.95	A:MET 19[N]	17	B:GLU 111[OE1]	3.55	A:LYS 61[NZ]
18	B:GLU 342[OE1]	3.06	A:GLN 190[NE2]	18	B:ASP 134[OD1]	3.21	A:LYS 181[NZ]
				19	B:ASP 134[OD2]	3.12	A:LYS 181[NZ]
				20	B:TYR 320[O]	2.81	A:GLN 183[NE2]
				21	B:GLU 342[OE1]	3.77	A:GLN 190[NE2]
				22	B:GLU 70[OE2]	2.85	A:ALA 259[N]
				23	B:GLU 57[OE1]	2.90	A:HIS 264[NE2]

Salt-Bridges

#	Chain A		Chain B
1	B:ARG 264[NH1]	3.87	A:GLU 57[OE1]
2	B:ARG 264[NH2]	3.34	A:GLU 57[OE1]
3	B:GLU 57[OE2]	2.68	A:ARG 264[NH1]
4	B:GLU 57[OE2]	2.47	A:ARG 264[NH2]

24	B:THR 21[O]	2.84	A:GLN 317[NE2]
25	B:MET 19[O]	2.64	A:SER 331[OG]

Salt-Bridges

#	Chain A	Distance [Å]	Chain B
1	B:LYS 61[NZ]	3.66	A:GLU 70[OE2]
2	B:LYS 181[NZ]	2.95	A:ASP 134[OD1]
3	B:LYS 181[NZ]	2.75	A:ASP 134[OD2]
4	B:HIS 264[NE2]	2.97	A:GLU 57[OE1]
5	B:GLU 70[OE2]	3.88	A:LYS 61[NZ]
